# Identification of a Non-Pentapeptide Region Associated with Rapid Mycobacterial Evolution

**DOI:** 10.1371/journal.pone.0154059

**Published:** 2016-05-05

**Authors:** Per Warholm, Sara Light

**Affiliations:** 1 Department of Biochemistry and Biophysics, Science for Life Laboratory, Stockholm University, SE-12 171 Solna, Sweden; 2 Department of Biochemistry and Biophysics, Bioinformatics Infrastructure for Life Sciences, Science for Life Laboratory, Stockholm University, SE-12 171 Solna, Sweden; Infectious Disease Research Institute, UNITED STATES

## Abstract

A large portion of the coding capacity of *Mycobacterium tuberculosis* is devoted to the production of proteins containing several copies of the pentapeptide-2 repeat, namely the PE/PPE_MPTR proteins. Protein domain repeats have a variety of binding properties and are involved in protein-protein interactions as well as binding to other ligands such as DNA and RNA. They are not as common in prokaryotes, compared to eukaryotes, but the enrichment of pentapeptide-2 repeats in *Mycobacteria* constitutes an exception to that rule. The genes encoding the PE/PPE_MPTR proteins have undergone many rearrangements and here we have identified the expansion patterns across the *Mycobacteria*. We have performed a reclassification of the PE/PPE_MPTR proteins using cohesive regions rather than sparse domain architectures. It is clear that these proteins have undergone large insertions of several pentapeptide-2 domains appearing adjacent to one another in a repetitive pattern. Further, we have identified a non-pentapeptide motif associated with rapid mycobacterial evolution. The sequence composition of this region suggests a different structure compared to pentapeptide-2 repeats. By studying the evolution of the PE/PPE_MPTR proteins, we have distinguished features pertaining to tuberculosis-inducing species. Further studies of the non-pentapeptide region associated with repeat expansions promises to shed light on the pathogenicity of *Mycobacterium tuberculosis*.

## Introduction

Protein domains are structural, functional and evolutionary building blocks that can form various architectures consisting of one or several domains [1, 2]. Protein domain repeats are strings of the same class of domain repeated one after another—tandem repeats. These domains are often short and their sequences are highly diverse, where typically only a motif is retained.

Proteins evolve through mutations involving one or a few residues and by domain rearrangements. The latter are comparatively well tolerated since, in many cases, protein domains perform modular functions. Repeat proteins have high variability with regard to the number of repeats in the protein. They differ from other proteins in the sense that they tend to expand through internal duplications rather than domain shuffling [3]. A likely scenario is that repeat proteins expand rapidly until a physical/structural limit has been reached and subsequently diverge rapidly since repeat domains tend to only have weak sequence similarity [3]. One possible explanation for their propensity is that their structures allow expansion and, additionally, may provide novel ligand binding [4].

Large expansions of protein repeats are quite common [5] and even commonplace in higher eukaryotes while they are rare in prokaryotes. However, one medically important example of large repeat expansions in prokaryotes are the events that have taken place within the pentapeptide-2 (PP2) proteins in *Mycobacteria* [6]. The PP2 repeat consists of five amino acids that are repeated one after the other in the proteins. The structure of the pentapeptide-2 repeat remains unknown, but its sister protein families [7] form four-sided parallell beta-helices. In *Mycobacteria*, there are predominantly two classes of proteins that contain PP2 repeats, namely the PE family (Proline-Glutamic Acid) and the PPE (Proline-Proline-Glutamic Acid) family. The number of repeats varies significantly between groups.

*M. tuberculosis is a pathogen that has limited genetic diversity and has likely emerged from more diverse strains by gaining virulence mechanisms [8] and the PPE_MPTR proteins are likely candidates for these emerging phenotypes. Along with its sister group, the PE_PGRS proteins, the PPE_MPTR proteins have been shown to be secreted through the ESX-5 (one of the secretion VII systems), probably affecting macrophage response [9, 10]. Furthermore, the PGRS and MPTR families have expanded along with duplications of the ESX-5 gene clusters, further supporting their importance for host response [11]*. Here, we elucidate the evolutionary path that has created the plethora of PP2 repeats that can be found in the PPE_MPTR proteins of *Mycobacteria*. By understanding the evolution of these proteins we can continue to elucidate the role of PP2 repeats for mycobacterial pathogenicity.

## Methods

### Sequence datasets

The protein sequences and genomes were downloaded from Genbank in 2015 [12].

### Domain assignment

The domain assignments were performed using HMMScan from the HMMER 3.0 software package [13] and Pfam-A release 27.0 [14] using an e-value of 10^−3^. Additional neighboring repeating domains were allowed a more relaxed e-value of 0.1, as previously described [15, 16]. The domain with the lowest e-value was selected when domains overlapped.

### Motif detection

Predicted Pfam domains using the e-values descried above were masked. Sequence stretches not containing any known domains were scanned for conserved motifs with MEME (Multiple Em for Motif Elicitation) from the MEME Suite software package [17]. Detected motifs could then be mapped to the dataset with the MAST (Motif Alignment & Search Tool) [18].

### Region classification

Even using the relaxed e-value cutoff, the primary domain assignment missed obvious repeat domains due to varied domain lengths. The HMM covers eight repeat units but we were able to identify shorter and longer units by manual inspection. To identify regions of repeats we used the HMM profile for all four known pentapeptides using a sliding window along the entire sequence. A residue is classified as belonging to the HMM with the highest positive score. Regions with scores less than zero were classified as non-pentapeptide regions.

We named the unmapped regions between the predicted domain regions “spacer regions”. Finally, a heuristic filter was used to concatenate adjacent regions that were too short to be filled by all eight repeat units. Using the method described above, we automatically classified all proteins in the dataset as (i) PPE, (ii) PP2 and (iii) spacer regions. The results are listed in S1 Table.

### The phylogenetic trees

Protein sequences containing repeating pentapeptide-2 (PF01469) domains and an N-terminal PPE domain (PF00823) were aligned using MUSCLE [19] with 16 iterations. Only the PPE domain is used to avoid bias due to repeat related length variation.

Phylogenetic and molecular evolutionary analyses were conducted using MEGA version 6 [20]. Phylogenetic trees were generated using maximum likelihood with a Jones-Taylor-Thornton [21] amino acid substitution matrix, gamma-distributed rate variation and a proportion of invariant sites with frequencies, using 500 bootstrap replicates.

### HMM-HMM dotplots

HMM-HMM dotplots were created using a modified version of HHalign [22] combined with a Python script utilizing NumPy [23]. The signal between similar amino acids is amplified using a moving average along the diagonal. Similarity below a threshold value of 0 was deemed insignificant and therefore removed. Finally, the similarity is normalized to values ranging from 0 and 1, where 0 is represented by white and 1 represented by black. The predicted Pfam domains were added above the plot using the same coloring scheme as in the phylogenetic tree. An accompanying autocorrolation plot was created by summing up and normalizing the diagonals. Cassette duplications show up as peaks in the plot—this is a pattern that makes the pentapeptide-2 repeat unique in comparison to other protein repeats (manuscript in preparation).

## Results and Discussion

**Fig 1:**
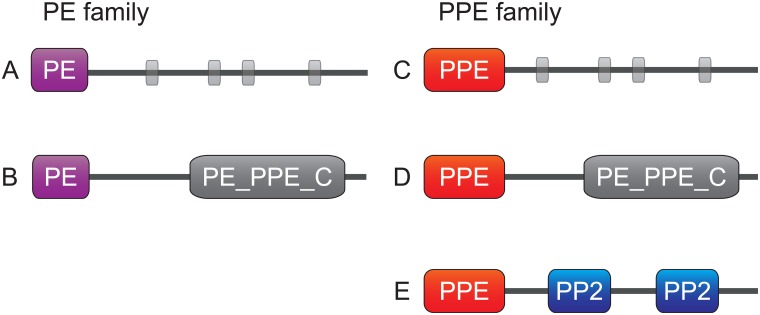
Schematic overview of common domain architectures in the PE/PPE protein family. A) A single N-terminal PE (Proline-Glutamate) domain followed by repeats of low complexity regions (grey boxes). Low complexity tails are also found in combination with a N-terminal PPE (Proline-Proline-Glutamate) domain (subfigure C). B) and D) share a C-terminal PE_PPE_C domain at the C-terminus. E) PPE_MPTR (Major Polymorphic Tandem Repeats) has a varied number of pentapeptide-2 (PP2) domains at the C-terminus.

The PE/PPE gene family encodes for genetically heterogeneous surface proteins [24] that are common in pathogens but largely missing in non-pathogenic bacteria [25, 26], see Fig 1 for a schematic illustration of the different members of this diverse group. PE/PPE proteins can affect immune evasion, antigenic variation and virulence [25] as they are expressed abundantly during infection [27]. The PP2 domains of the proteins of the PPE_MPTR family have been identified as being primarily responsible for eliciting a humoral immune response in patients with relapsed tuberculosis [28].

Despite its importance as a probable player in pathogenicity, the body of literature covering the PPE_MPTR proteins is quite small compared to the PE_PGRS proteins. A PubMed (PMC) search for “PGRS AND PE AND MYCOBACTERIUM” generates 379 publications while the same search for the MPTR family generates 29 publications.

Due to its importance for pathogenicity it is not surprising that the PE/PPE family evolves rapidly through a number of different genetic mechanisms. First, the gene family exhibits high synonymous and nonsynonymous substitution frequencies [29]. Second, PE/PPE proteins are hotspots for insertions of transposable elements as well as other types of recombination events [30]. In fact, the PE/PPE proteins may be a major source of antigenic variation by means of inter-strain polymorphism [31]. Finally, microsatellite polymorphism is another driving force for mycobacterial genomic plasticity [32].

### Pentapeptide-2 repeat expansions in *Mycobacteria*

The pentapeptide-2 (PP2) domain is only abundant in *Mycobacteria* (95% of all PP2 sequences in Pfam [7] originate from *Mycobacteria*). We extracted a dataset with 141 proteins containing PP2 repeats. The dataset is dominated by proteins from the tuberculosis-causing pathogens *Mycobacterium africanum*, *Mycobacterium bovis*, *Mycobacterium canettii* and *Mycobacterium tuberculosis*.

The pentapeptide-2 repeats may form a right-handed *β*-helical structure [33]. Inspection of the domain architecture shows that an overwhelming majority of these proteins also contain an N-terminal domain, the PPE (Proline-Proline-Glutamic Acid) domain, that is unique to *Mycobacteria* and particularly common in mycobacterial pathogens [11]. This protein constitutes the PPE-MPTR (major polymorphic tandem repeat) subfamily [34].

**Fig 2:**
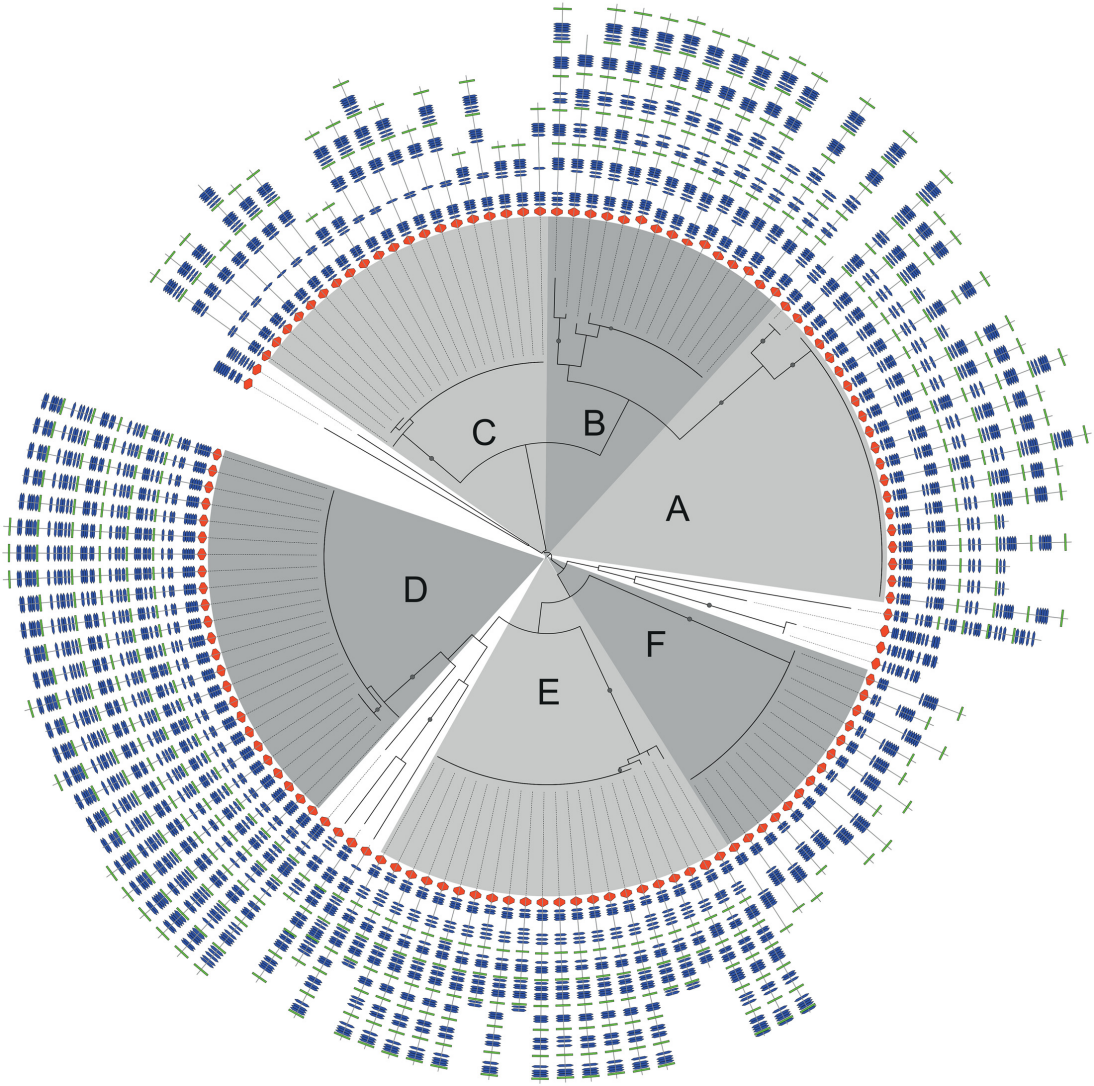
Phylogenetic tree showing the evolutionary relationship between protein sequences containing both pentapeptide-2 domain (PF01469) repeats and a PPE domain (PF00823). The domain architecture is shown in the outer circle where the red horizontal hexagon represents the PPE domain, blue vertical hexagons are pentapeptide-2 domains, the green rectangles are a conserved motif (see S1 Fig) that we use to trace cassette expansions. Edges with a bootstrap value above 80 are marked with a dot. Large groups, labeled A to F, are marked with grey background color.

**Fig 3:**
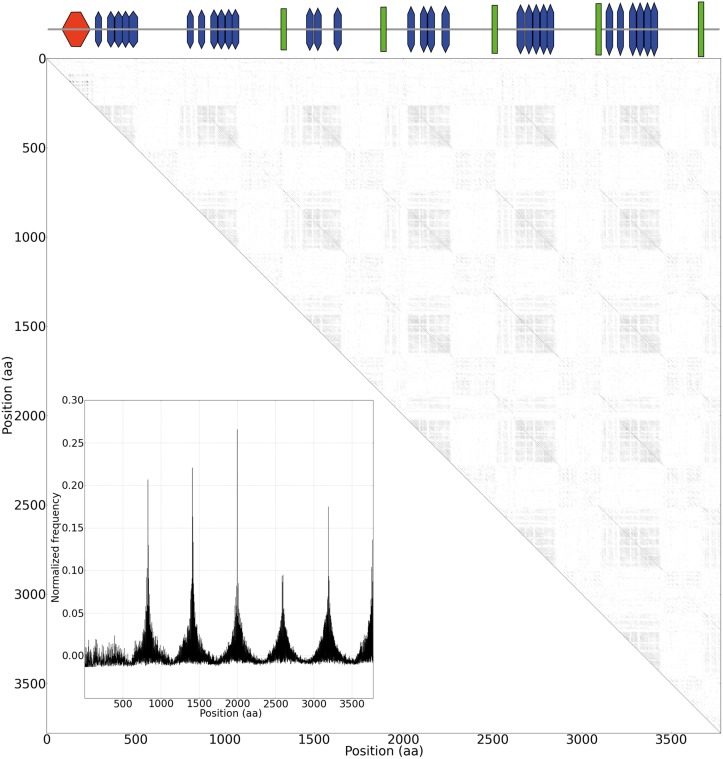
Internal similarity of the longest pentapeptide-2 repeat protein. A HMM-HMM dotplot with the longest protein (GI:148824559) containing pentapeptide-2 repeats (Pfam: PF01469) compared against itself. Darker dots indicate higher sequence similarity. The domain architecture is shown on top. Red is the PPE family (PF00823), blue is pentapeptide-2 repeats (PF01469), green is a conserved motif (see S2 Fig). (B) Autocorrelation plot of the data shown in (A) It visualizes the clear similarity between the cassette expansions. Six peaks indicate that six recent expansions have taken place. The cassette similarity can be seen as diagonal lines in the dotplot in (A).

In Fig 2 a domain—centric evolutionary analysis of the PP2-containing proteins is presented. The longest protein in the dataset is shown as an example in Fig 3. Here, duplications of cassettes of repeat domains are visible as periodic dark blocks in the HMM-HMM similarity dotplots. Such duplications will be referred to as cassette duplications. In a strikingly large number of cases there are tandem duplications within these 141 proteins, as can be seen in S1 Fig, where large cassettes of smaller repeats have been inserted.

### Duplications of Pentapeptide-2 repeats are associated with a spacer region

One interesting aspect that can immediately be gleaned from Fig 2 is that the cassette events have occurred in the proteins with sparse (rather than compact) domain architectures. For lack of a better term we will refer to these regions as *spacer regions*. These spacer regions show up as weaker blocks between the PP2 blocks, see Fig 3. The high similarity between the cassettes is seen through the periodic diagonals and as seven high peaks in the embedded autocorrolation plot in Fig 3.

In addition to the spacer regions and the PP2 domains we were able to identify a recurring motif, *event motif*, in the spacer regions, see S2 Fig. Detected event motifs are located in the regions bordering the PP2 regions. Given that all but one cassette event are associated with the event motif, as shown in Fig 2, we can surmise that the motif is either (i) of functional importance or (ii) a conserved repeat, perhaps associated with slipped strand mispairing occurs. Inspection of the DNA-alignments of these sequences indicates that the motif is not conserved at the DNA level. Therefore, this motif may be of functional importance and this rapidly evolving subset of the PPE proteins could play a role in mycobacterial virulence.

### Inherent division into evolutionary groups

The larger groups contain proteins of different lengths due to cassette expansions. The *event motif*, only present where cassette duplications have occured, enables us to identify the order of the cassette expansions.

Six larger groups (labeled A to F) were identified, see Fig 2. Proteins that are evolutionarily dissimilar were marked with Z in Table 1. Proteins clustered in the same group have similar domain architectures, despite length variation.

**Table 1:** Number of proteins present in phylogenetic groups for strains of Mycobacterium.

	Phylogenetic group								
Species	A	B	C	D	E	F	Z	Num	Total
Mycobacterium africanum GM041182	0	0	1	0	0	1	0	0	2
Mycobacterium bovis AF2122/97	1	0	1	1	1	1	0	1	6
Mycobacterium bovis BCG str. Korea 1168P	1	0	1	1	1	0	0	3	7
Mycobacterium bovis BCG str. Mexico	1	0	1	1	1	1	0	1	6
Mycobacterium bovis BCG str. Pasteur 1173P2	1	0	1	1	1	1	0	1	6
Mycobacterium bovis BCG str. Tokyo 172	1	0	1	1	1	1	0	1	6
Mycobacterium canettii CIPT 140010059	1	1	1	1	1	0	0	0	5
Mycobacterium canettii CIPT 140060008	1	1	0	1	1	1	0	0	5
Mycobacterium canettii CIPT 140070008	1	0	0	1	1	0	0	0	3
Mycobacterium canettii CIPT 140070010	1	0	0	1	1	0	1	1	5
Mycobacterium canettii CIPT 140070017	0	2	0	1	1	0	1	0	5
Mycobacterium marinum M	0	0	0	0	0	0	7	0	7
Mycobacterium tuberculosis 7199-99	1	1	1	1	1	1	0	1	7
Mycobacterium tuberculosis CCDC5079	0	0	1	1	1	0	0	2	5
Mycobacterium tuberculosis CCDC5180	1	1	1	1	1	1	0	0	6
Mycobacterium tuberculosis CDC1551	0	0	0	1	1	0	0	0	2
Mycobacterium tuberculosis CTRI-2	1	1	1	1	1	1	0	0	6
Mycobacterium tuberculosis F11	0	1	1	1	1	0	0	1	5
Mycobacterium tuberculosis H37Ra	1	1	1	1	0	0	0	1	5
Mycobacterium tuberculosis H37Rv	2	2	2	2	0	0	0	2	10
Mycobacterium tuberculosis KZN 1435	1	1	1	1	1	1	0	0	6
Mycobacterium tuberculosis KZN 4207	1	1	1	1	1	1	0	0	6
Mycobacterium tuberculosis KZN 605	1	1	1	1	1	1	0	0	6
Mycobacterium tuberculosis UT205	0	0	0	1	1	1	0	0	3
Mycobacterium tuberculosis str. Beijing/NITR203	1	0	1	0	1	0	0	0	3
Mycobacterium tuberculosis str. Erdman = ATCC 35801	1	1	1	1	1	1	0	1	7
Mycobacterium ulcerans Agy99	0	0	0	0	0	0	1	0	1

Table notes: Ungrouped proteins are labeled Z. The number of PP2 repeat proteins lacking a PPE domain is listed in the Num column.

Inspection of the sequences indicates that expansions appear to have arisen multiple times independently. Shorter sequences display different behavior where new domains have been added consecutively forming a cluster pattern (indicating that one domain at a time has been duplicated) instead of large cassettes.

A close-up of group A shows that there is substantial length variation between closely related proteins. The proteins of *M. bovis* have four cassettes, *M. tubeculosis*’s proteins have five and the proteins of *M. canettii* either two two or six cassettes.

All proteins in groups A, B, D and E contain alternating PP2 and spacer regions. Groups C and F exhibit a different pattern where there is only one *event motif* and the proteins are more sparsely populated with regard to domain assignments.

### Tuberculosis-inducing strains have a different distribution of PE/PPE proteins

In Table 1, all *M. bovis* proteins are homogenous with each strain missing proteins from phylogenetic group B. The only strain missing a protein in group F has two additional proteins without a PPE domain.

The various strains of *M. tuberculosis* and *M. canettii* show more variation. Although *M. canettii* is closely related to *M. tuberculosis* it has a different morphology and shorter generation time compared to some other tuberculosis-causing strains [35]. This can also be seen in the phylogenetic tree, where the proteins appear in subclusters seperated from *M. bovis* and *M. tuberculosis*.

*M. africanum* only has proteins belonging to groups C and F. While *M. africanum* causes tuberculosis, it has a lower rate of progression [36], possibly related to the lack of proteins in groups A, B, D and E.

The non-tuberculosis-inducing species *M. marinum* and *M. ulcerans* have exclusively non-grouped proteins (Group Z).

### Regions rather than protein domains

**Fig 4:**
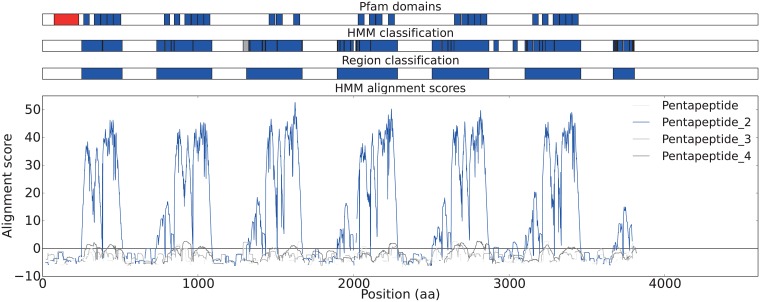
Automatic classification of regions of the pentapeptide-2 repeat protein GI:148824559. The line plot shows HMM alignment score between the protein sequence and HMM profiles for four different pentapeptide repeats. The top track shows predicted Pfam-A domains, red is PPE (PF00823) and blue is Pentapeptide-2 (PF01469). The regions in the second track are based of the best fitting HMM score. The third track with pentapeptide-2 region classification is determined by applying a heuristic filter to the second track.

Short repetitive elements of varying length are difficult to map to discrete positions in the sequence. By using larger continuous regions this issue can be avoided. Automatic classification divides the proteins into PPE, PP2 and spacer regions. An example for the longest protein in the data set is shown in Fig 4. The comprehensive result can be seen in S1 Table. A visual representation of the region data can be found in the supplementary material (S6 Fig). *M. tuberculosis* has more regions than *M. bovis*. *M. canettii* has the proteins with the most regions, but also copies with fewer regions.

#### The PPE region

The PPE domain appears in the N terminus in 125 out of 141 proteins. It is much longer than the pentapeptide-2 repeats (about 180 aa) and well conserved. A histogram with region size shows that there is little length variation in the PPE domain.

#### The pentapeptide-2 region

The Pfam domain is defined as eight repeats of five (XNXGX), i.e. a 40 aa long repeat domain, see S3 Fig. With longer stretches containing multiple repeats of repeats it is not trivial to define the starts and ends of the repeat regions. Automatic classification of the regions can be done using Pfam HMM-profiles and a sliding window (along the protein sequence). This approach allows identification of parts of the sequences that would otherwise remain unclassified.

#### The spacer region

**Fig 5:**
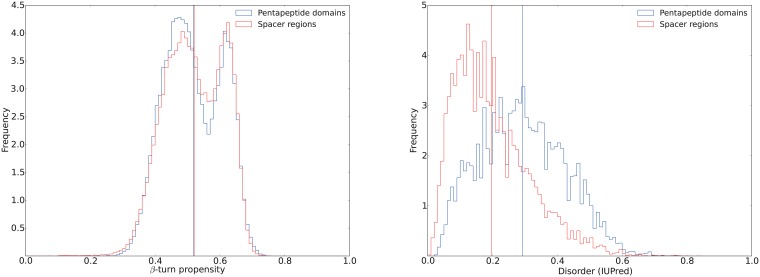
Pentapeptide-2 and spacer region similarity and dissimilarity. (A) A histogram of *β*-turn propensity for all residues in pentapeptide-2 domains (blue) and spacer regions (red) between the pentapeptide-2 domains. The means are shown as vertical lines at 0.52. (B) A histogram of disorder for all residues in pentapeptide-2 domains (blue) and spacer regions (red).

We identified the spacer regions due to a recurring pattern devoid of Pfam domains, inbetween PP2 domains. Detailed analysis indicates that the spacer regions have similar attributes when it comes to structure but poor sequence similarity. Spacer regions come in different sizes with two peaks around 100 and 220 amino acids. The complete size distribution can be seen in histogram, S4 Fig. Like the PP2 domains, spacer regions are predominately predicted as random coils. When comparing the spacer regions with the PP2 regions there is no difference between the predicted propensity to form *β*-turns (Fig 5 panel A). The former, however, are much less disordered (Fig 5 panel B) due to a different amino acid distribution with considerably fewer glycines. There is a large difference in disorder between the Pentapeptide-2 and the spacer regions. There is no correlation (Pearson’s correlation coefficient: 0.01, 2-tailed p-value: 0.89) between disorder and protein length.

### The spacer region is not a pentapeptide repeat

**Fig 6:**
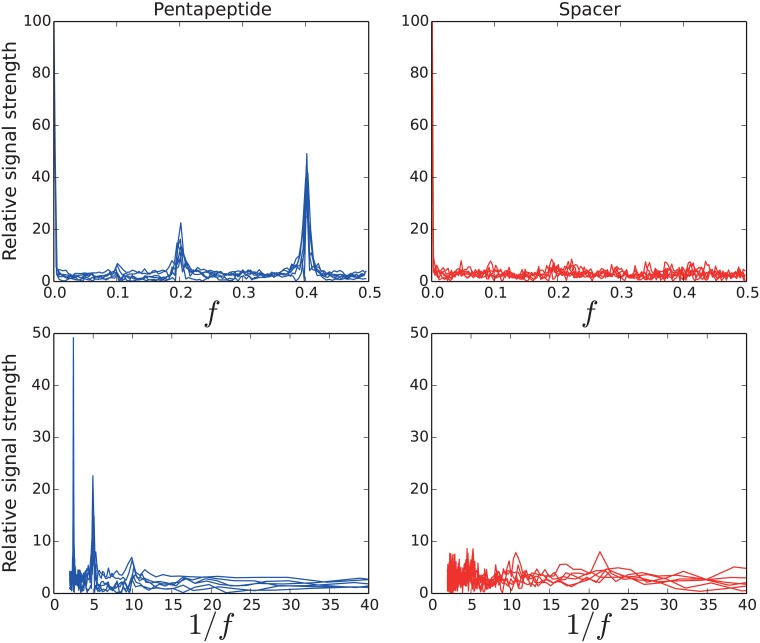
Internal repeats size detected through Fast Fourier transform. A peak in the Fourier wavespace corresponds to a repeating signal. Pentapeptide has a clear peak at 5 as expected (2-tailed p-value: 3.94×10^−3^). There are also peaks at half (2.5) and double (10) that size. The spacer regions have weaker peaks at around 5, 11 and 22 although they are not statistically significant.

The pentapeptide repeats in the PP2 regions can be detected using Fast-Fourier transform (see Fig 6) of the HMM-profile similarity score. This method is commonly used to detect periodic signals [37]. The same signal, with repeats of five and ten amino acids, is not seen in the spacer regions. While it is possible that the PP2 repeat has a repeated structure, perhaps of a quadrilateral beta-helix where each turn is five amino acids long, it is likely that the spacer regions have a completely different structure.

## Conclusions

In the prokaryotes, long repeat proteins are comparatively rare. Nonetheless, there are considerable repeat expansions in certain prokaryotic lineages. Mutation rates in bacteria are non-randomly distributed across the genomes [38] and it has been suggested that such lineage-specific gene families may be of adaptive significance [39].

We have found that the pentapeptide-2 repeats in the PE/PPE genes of *Mycobacteria* expand through single duplication events that encompass several domains (cassette duplications), possibly facilitated through homologous recombination which, although thought to be rare in *Mycobacteria* [40], is enriched in PE/PPE regions [41]. The PE/PPE regions are also known to exhibit relaxed selective constraints [42] and rapid evolutionary rates [43].

We have extracted a motif that is strongly linked to the proteins where large duplications have occurred. This motif is conserved at the amino acid level but not conserved at the DNA level and is therefore possibly of functional importance. The pentapeptide-2 repeat expansions also illustrate the malleable nature of prokaryotic protein domains, as the motif constitutes an elaboration upon the pentapeptide-2 domain. Indeed, *Mycobacteria* lack the post-replicative mismatch repair system [44], an omission that may lead to repeat sequence diversity [45].

The PPE proteins of *Mycobacteria* have long been recognized as an important group of proteins that may be responsible for much of the surface variability of *Mycobacterium tuberculosis*[6]. Considering the proposed role of these proteins for mycobacterial pathogenicity, further *in vivo* studies of the motif we have identified as associated with repeat expansions may elucidate the mechanisms behind the rapidly emerging pathogenic arsenal of *Mycobateria*.

## Supporting Information

S1 Fig Cassette duplications are common in PE/PPE proteins. Six protein similarity dotplots, each from one of groups, A to F, in Fig 1. A darker color indicates higher similarity. When larger parts are duplicated they show up as repeated dark blocks.
(EPS)

S2 Fig Logo for the motif associated with duplication events in the pentapeptide proteins in *Mycobacteria*. (PDF)

S3 Fig Logo for the pentapeptide-2 domain. Adapted from Pfam, PF01469. Vertical lines have been added after every fifth position.
(PNG)

S4 Fig Histogram showing the lengths distribution of regions classified as PPE, Pentapeptide-2 and Spacer. (PNG)

S5 Fig Long pentapeptide-2 repeat proteins have less beta-turn propensity. There is a very strong negative correlation (Pearson’s correlation coefficient: −0.90, 2-tailed p-value: 1.11×10^−52^) between *β*-turn propensity and protein length. The PPE domain in the N-terminal doesn’t form a *β*-helix and the pattern is even more clear when only the sequence downstream of the PPE domain is considered.
(PNG)

S6 Fig The number of PP2 and spacer regions in various Mycobacterium species. A visual representation of the data in S1 Table, showing the number of PP2 and spacer regions for each protein in the dataset. Species are represented by different colors. Strains have different markers.
(EPS)

S1 Table Number of regions for each protein in the dataset. determined using a automatic classification script based on HMM alignment scores. The table is sorted by species and protein name.
(PDF)

S1 Dataset Pentapeptide-2 dataset. A collection of data concerning the pentapeptide-2 proteins described in S1 Table.
(7Z) Click here for additional data file.
