# Supplementary figures and images for: Identification of a Non-Pentapeptide Region Associated with Rapid Mycobacterial Evolution
Source: PLoS One. 2016 May 5;11(5):e0154059. doi: 10.1371/journal.pone.0154059 (PMC4858275; doi:10.1371/journal.pone.0154059)

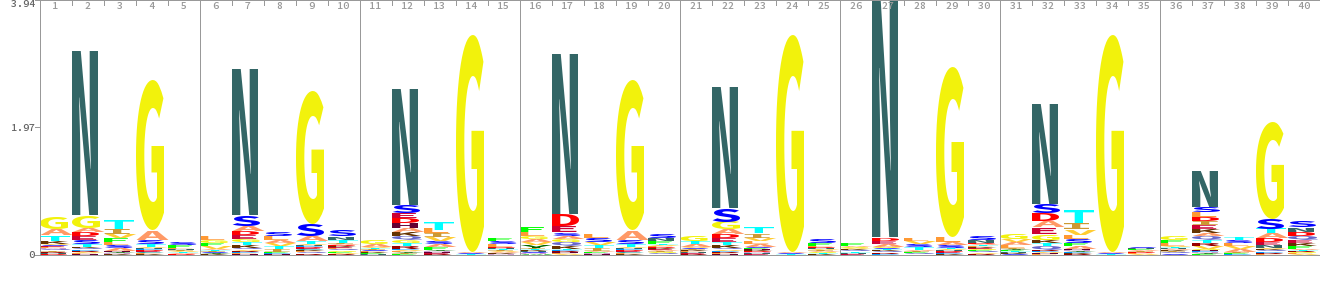

Supplement: S3 Fig — Adapted from Pfam, PF01469. Vertical lines have been added after every fifth position. (PNG) [file pone.0154059.s003.png]

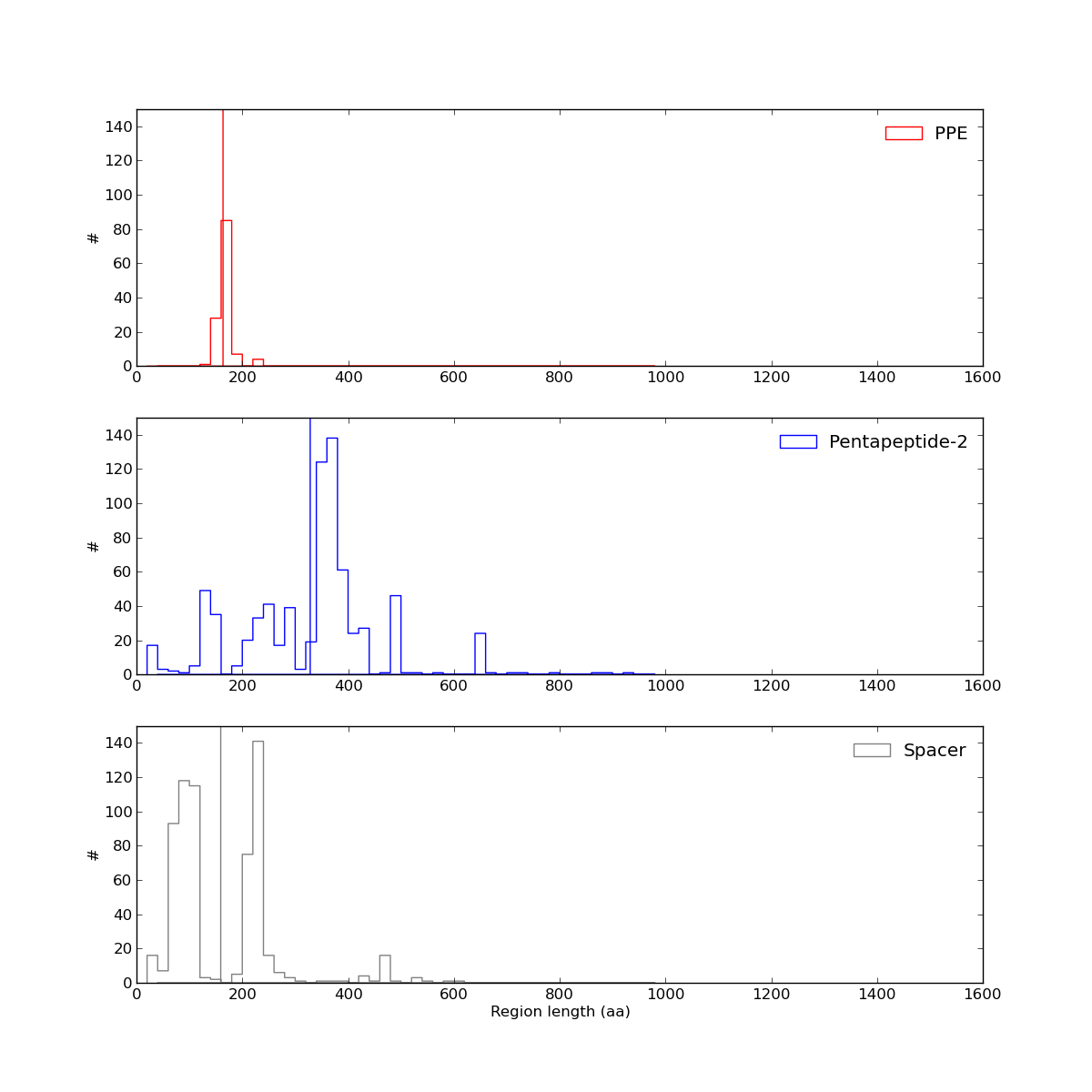

Supplement: S4 Fig — (PNG) [file pone.0154059.s004.png]

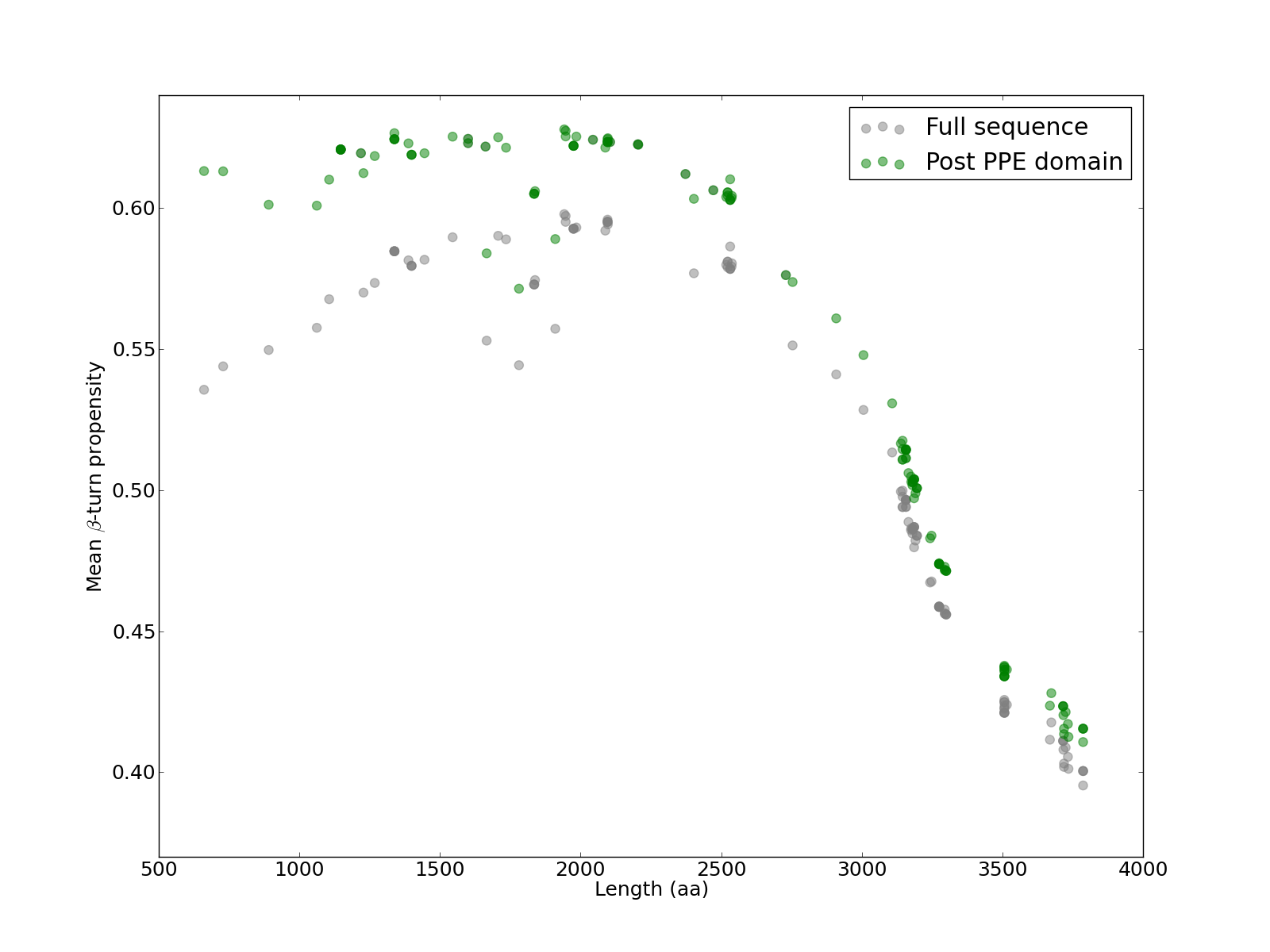

Supplement: S5 Fig — There is a very strong negative correlation (Pearson’s correlation coefficient: −0.90, 2-tailed p-value: 1.11×10^−52^) between β-turn propensity and protein length. The PPE domain in the N-terminal doesn’t form a β-helix and the pattern is even more clear when only the sequence downstream of the PPE domain is considered. (PNG) [file pone.0154059.s005.png]
