# Supplementary material for: Identification of a Non-Pentapeptide Region Associated with Rapid Mycobacterial Evolution
Source: PLoS One. 2016 May 5;11(5):e0154059. doi: 10.1371/journal.pone.0154059 (PMC4858275; doi:10.1371/journal.pone.0154059)
Supplement: S1 Table — determined using a automatic classification script based on HMM alignment scores. The table is sorted by species and protein name. (PDF) [file pone.0154059.s007.pdf]

Table S1

Number of region for each protein in the dataset determined using a automatic classification script based on HMM alignment scores. The table is sorted by species and protein name.

		Region count			
**GI Number**	**Species**	**PPE**	**PP2**	**Spacer**	**Phylo group**
339631970	Mycobacterium africanum GM041182	1	3	2	F
339633354	Mycobacterium africanum GM041182	1	6	5	C
31791483	Mycobacterium bovis AF2122/97	0	3	2	Num
31791484	Mycobacterium bovis AF2122/97	1	5	4	E
31791532	Mycobacterium bovis AF2122/97	1	7	6	D
31793109	Mycobacterium bovis AF2122/97	1	3	2	F
31794527	Mycobacterium bovis AF2122/97	1	3	2	C
31794530	Mycobacterium bovis AF2122/97	1	4	4	A
449062300	Mycobacterium bovis BCG str. Korea 1168P	0	3	2	Num
449062301	Mycobacterium bovis BCG str. Korea 1168P	1	5	4	E
449062349	Mycobacterium bovis BCG str. Korea 1168P	1	7	6	D
449065454	Mycobacterium bovis BCG str. Korea 1168P	1	3	2	C
449065459	Mycobacterium bovis BCG str. Korea 1168P	1	4	4	A
449065463	Mycobacterium bovis BCG str. Korea 1168P	0	3	2	Num
449065464	Mycobacterium bovis BCG str. Korea 1168P	0	4	5	Num
378770052	Mycobacterium bovis BCG str. Mexico	0	3	2	Num
378770053	Mycobacterium bovis BCG str. Mexico	1	5	4	E
378770101	Mycobacterium bovis BCG str. Mexico	1	7	6	D
378771665	Mycobacterium bovis BCG str. Mexico	1	3	2	F
378773132	Mycobacterium bovis BCG str. Mexico	1	3	2	C
378773135	Mycobacterium bovis BCG str. Mexico	1	4	4	A
121636219	Mycobacterium bovis BCG str. Pasteur 1173P2	0	3	2	Num
121636220	Mycobacterium bovis BCG str. Pasteur 1173P2	1	5	4	E
121636268	Mycobacterium bovis BCG str. Pasteur 1173P2	1	7	6	D
121637822	Mycobacterium bovis BCG str. Pasteur 1173P2	1	3	2	F
121639271	Mycobacterium bovis BCG str. Pasteur 1173P2	1	3	2	C
121639274	Mycobacterium bovis BCG str. Pasteur 1173P2	1	4	4	A
224988692	Mycobacterium bovis BCG str. Tokyo 172	0	3	2	Num
224988693	Mycobacterium bovis BCG str. Tokyo 172	1	5	4	E
224988740	Mycobacterium bovis BCG str. Tokyo 172	1	7	6	D
224990306	Mycobacterium bovis BCG str. Tokyo 172	1	3	2	F
224991768	Mycobacterium bovis BCG str. Tokyo 172	1	3	2	C
224991771	Mycobacterium bovis BCG str. Tokyo 172	1	4	4	A
340625336	Mycobacterium canettii CIPT 140010059	1	7	6	E
340625385	Mycobacterium canettii CIPT 140010059	1	7	6	D
340628327	Mycobacterium canettii CIPT 140010059	1	6	5	C
340628330	Mycobacterium canettii CIPT 140010059	1	8	7	A
340628332	Mycobacterium canettii CIPT 140010059	1	7	6	B
433625404	Mycobacterium canettii CIPT 140060008	1	7	6	E
433625450	Mycobacterium canettii CIPT 140060008	1	7	6	D
433627012	Mycobacterium canettii CIPT 140060008	1	3	2	F
433628492	Mycobacterium canettii CIPT 140060008	1	7	6	A
433628495	Mycobacterium canettii CIPT 140060008	1	7	6	B
433640434	Mycobacterium canettii CIPT 140070008	1	7	6	E

433640476	Mycobacterium canettii CIPT 140070008	1	7	6	D
433643545	Mycobacterium canettii CIPT 140070008	1	7	7	A
433629402	Mycobacterium canettii CIPT 140070010	1	7	6	E
433629445	Mycobacterium canettii CIPT 140070010	1	7	6	D
433629998	Mycobacterium canettii CIPT 140070010	1	4	3	Z
433632448	Mycobacterium canettii CIPT 140070010	0	5	5	Num
433632452	Mycobacterium canettii CIPT 140070010	1	2	2	A
433633322	Mycobacterium canettii CIPT 140070017	1	7	6	E
433633365	Mycobacterium canettii CIPT 140070017	1	1	0	Z
433633369	Mycobacterium canettii CIPT 140070017	1	7	6	D
433636452	Mycobacterium canettii CIPT 140070017	1	7	6	B
433636455	Mycobacterium canettii CIPT 140070017	1	7	6	B
183980666	Mycobacterium marinum M	1	5	4	Z
183980667	Mycobacterium marinum M	1	6	5	Z
183981653	Mycobacterium marinum M	1	5	4	Z
183983527	Mycobacterium marinum M	1	1	0	Z
183984290	Mycobacterium marinum M	1	1	0	Z
183984588	Mycobacterium marinum M	1	7	6	Z
183985299	Mycobacterium marinum M	1	1	0	Z
479054243	Mycobacterium tuberculosis 7199-99	1	7	6	E
479054293	Mycobacterium tuberculosis 7199-99	1	6	6	D
479055874	Mycobacterium tuberculosis 7199-99	1	3	2	F
479057321	Mycobacterium tuberculosis 7199-99	1	5	4	C
479057325	Mycobacterium tuberculosis 7199-99	1	6	5	A
479057328	Mycobacterium tuberculosis 7199-99	0	4	3	Num
479057329	Mycobacterium tuberculosis 7199-99	1	4	3	B
385993408	Mycobacterium tuberculosis CCDC5079	1	4	4	E
385993456	Mycobacterium tuberculosis CCDC5079	1	6	6	D
385996217	Mycobacterium tuberculosis CCDC5079	1	6	6	C
385996220	Mycobacterium tuberculosis CCDC5079	0	5	5	Num
385996224	Mycobacterium tuberculosis CCDC5079	0	4	3	Num
385989814	Mycobacterium tuberculosis CCDC5180	1	7	6	E
385989862	Mycobacterium tuberculosis CCDC5180	1	6	6	D
385991283	Mycobacterium tuberculosis CCDC5180	1	3	2	F
385992585	Mycobacterium tuberculosis CCDC5180	1	7	6	C
385992588	Mycobacterium tuberculosis CCDC5180	1	6	5	A
385992591	Mycobacterium tuberculosis CCDC5180	1	7	6	B
15839689	Mycobacterium tuberculosis CDC1551	1	7	6	E
15839741	Mycobacterium tuberculosis CDC1551	1	6	6	D
385997079	Mycobacterium tuberculosis CTRI-2	1	7	6	E
385997129	Mycobacterium tuberculosis CTRI-2	1	6	6	D
385998693	Mycobacterium tuberculosis CTRI-2	1	3	2	F
386000137	Mycobacterium tuberculosis CTRI-2	1	6	5	C
386000141	Mycobacterium tuberculosis CTRI-2	1	6	5	A
386000144	Mycobacterium tuberculosis CTRI-2	1	7	6	B
148821501	Mycobacterium tuberculosis F11	1	7	6	E
148821551	Mycobacterium tuberculosis F11	1	6	6	D
148824549	Mycobacterium tuberculosis F11	1	6	5	C
148824553	Mycobacterium tuberculosis F11	0	5	4	Num
148824559	Mycobacterium tuberculosis F11	1	7	6	B

148660070	Mycobacterium tuberculosis H37Ra	0	5	5	Num
148660121	Mycobacterium tuberculosis H37Ra	1	6	6	D
148663206	Mycobacterium tuberculosis H37Ra	1	5	4	C
148663209	Mycobacterium tuberculosis H37Ra	1	6	5	A
148663212	Mycobacterium tuberculosis H37Ra	1	7	6	B
397672095	Mycobacterium tuberculosis H37Rv	0	5	4	Num
397672146	Mycobacterium tuberculosis H37Rv	1	6	6	D
397675290	Mycobacterium tuberculosis H37Rv	1	6	5	C
397675294	Mycobacterium tuberculosis H37Rv	1	6	5	A
397675297	Mycobacterium tuberculosis H37Rv	1	7	6	B
448824742	Mycobacterium tuberculosis H37Rv	1	6	6	D
57116719	Mycobacterium tuberculosis H37Rv	0	5	4	Num
57117092	Mycobacterium tuberculosis H37Rv	1	6	5	C
57117095	Mycobacterium tuberculosis H37Rv	1	6	5	A
57117096	Mycobacterium tuberculosis H37Rv	1	7	6	B
253797231	Mycobacterium tuberculosis KZN 1435	1	7	6	E
253797281	Mycobacterium tuberculosis KZN 1435	1	6	6	D
253799036	Mycobacterium tuberculosis KZN 1435	1	3	2	F
253800388	Mycobacterium tuberculosis KZN 1435	1	6	5	C
253800392	Mycobacterium tuberculosis KZN 1435	1	6	5	A
253800397	Mycobacterium tuberculosis KZN 1435	1	7	6	B
375294513	Mycobacterium tuberculosis KZN 4207	1	7	6	E
375294563	Mycobacterium tuberculosis KZN 4207	1	6	6	D
375296286	Mycobacterium tuberculosis KZN 4207	1	3	2	F
375297616	Mycobacterium tuberculosis KZN 4207	1	6	5	C
375297620	Mycobacterium tuberculosis KZN 4207	1	6	5	A
375297625	Mycobacterium tuberculosis KZN 4207	1	7	6	B
392430723	Mycobacterium tuberculosis KZN 605	1	7	6	E
392430773	Mycobacterium tuberculosis KZN 605	1	6	6	D
392432499	Mycobacterium tuberculosis KZN 605	1	3	2	F
392433827	Mycobacterium tuberculosis KZN 605	1	6	5	C
392433831	Mycobacterium tuberculosis KZN 605	1	6	5	A
392433836	Mycobacterium tuberculosis KZN 605	1	7	6	B
392385024	Mycobacterium tuberculosis UT205	1	7	6	E
392385073	Mycobacterium tuberculosis UT205	1	6	6	D
392386573	Mycobacterium tuberculosis UT205	1	3	2	F
479313161	Mycobacterium tuberculosis str. Beijing/NITR203	1	7	6	E
479316336	Mycobacterium tuberculosis str. Beijing/NITR203	1	6	5	C
479316342	Mycobacterium tuberculosis str. Beijing/NITR203	1	6	5	A
471336120	Mycobacterium tuberculosis str. Erdman = ATCC 35801	1	7	6	E
471336174	Mycobacterium tuberculosis str. Erdman = ATCC 35801	1	6	6	D
471337867	Mycobacterium tuberculosis str. Erdman = ATCC 35801	1	2	2	F
471339399	Mycobacterium tuberculosis str. Erdman = ATCC 35801	1	5	4	C
471339403	Mycobacterium tuberculosis str. Erdman = ATCC 35801	1	6	5	A
471339408	Mycobacterium tuberculosis str. Erdman = ATCC 35801	0	4	3	Num
471339409	Mycobacterium tuberculosis str. Erdman = ATCC 35801	1	4	3	B
118616725	Mycobacterium ulcerans Agy99	1	1	0	Z
