# Supplementary material for: Identification of a Non-Pentapeptide Region Associated with Rapid Mycobacterial Evolution
Source: PLoS One. 2016 May 5;11(5):e0154059. doi: 10.1371/journal.pone.0154059 (PMC4858275; doi:10.1371/journal.pone.0154059)
Supplement: S2 Fig — (PDF) [file pone.0154059.s002.pdf]

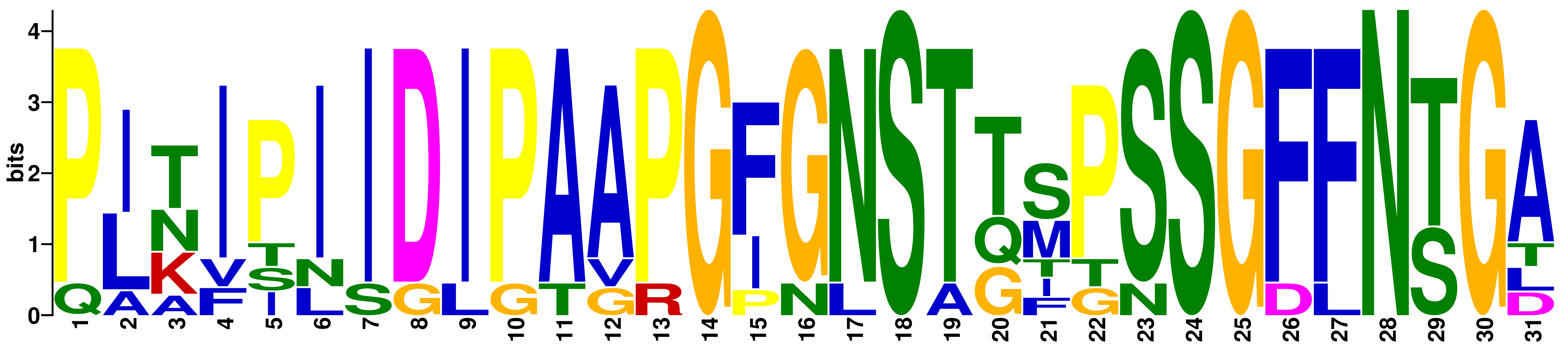
bits
MEME (no SSC) 27.11.13 13:00
